# Role of cholesterol in substrate recognition by $\gamma$-secretase

**DOI:** 10.1038/s41598-021-94618-2

**Published:** 2021-07-26

**Authors:** Łukasz Nierzwicki, Michał Olewniczak, Paweł Chodnicki, Jacek Czub

**Affiliations:** Department of Physical Chemistry, Gdansk University of Technology, Gdansk, 80-233 Poland

**Keywords:** Computational biophysics, Enzyme mechanisms, Proteases

## Abstract

$\gamma$-Secretase is an enzyme known to cleave multiple substrates within their transmembrane domains, with the amyloid precursor protein of Alzheimer’s Disease among the most prominent examples. The activity of $\gamma$-secretase strictly depends on the membrane cholesterol content, yet the mechanistic role of cholesterol in the substrate binding and cleavage remains unclear. In this work, we used all-atom molecular dynamics simulations to examine the role of cholesterol in the initial binding of a direct precursor of $\beta$-amyloid polypeptides by $\gamma$-secretase. We showed that in cholesterol-rich membranes, both the substrate and the enzyme region proximal to the active site induce a local membrane thinning. With the free energy methods we found that in the presence of cholesterol the substrate binds favorably to the identified exosite, while cholesterol depletion completely abolishes the binding. To explain these findings, we directly examined the role of hydrophobic mismatch in the substrate binding to $\gamma$-secretase, showing that increased membrane thickness results in higher propensity of the enzyme to bind substrates. Therefore, we propose that cholesterol promotes substrate binding to $\gamma$-secretase by increasing the membrane thickness, which leads to the negative hydrophobic mismatch between the membrane and binding partners.

## Introduction

$\gamma$-Secretase is an intramembraneaspartyl protease that is involved in regulated intramembrane proteolysis^1^. It cleaves multiple signalling proteins within their transmembrane domains (TMDs)^2,3^. The most prominent examples of $\gamma$-secretase substrates include amyloid precursor protein (APP), linked to the development of Alzheimer’s Disease^4^ and Notch protein responsible for cell proliferation and differentiation^5^. The aberrant processing of APP results in the excessive production of $\beta$-amyloid polypeptides, which aggregate into oligomeric assemblies that impair synaptic activity, as well as form neuritic plaques that cause neuronal death^6,7^. Likewise, defects in Notch signalling impair the maturation of the cells, contributing to the development of several types of cancer^8^.

$\gamma$-secretase comprises of four components: nicastrin, APH-1, PEN-2 and presenilin, with the latter one being the catalytic subunit of the enzyme^9^. The presenilin subunit is composed of 9 transmembrane helices (TMs), with the catalytic aspartates (D257 and D385) located at the TM6 and TM7, respectively^10^. Maturation of the $\gamma$-secretase complex involves autocatalytic cleavage between TM6 and TM7 of presenilin, which results in the production of the N- and C-terminal fragments (NTF and CTF) of the subunit^11^. Both before and after autoproteolysis, the catalytic residues are buried inside the enzyme and are shielded from the hydrophobic environment of the membrane^12^.

It has been suggested that access to the enzyme active site is regulated by a lateral cleft between the helices of presenilin^13^. Initially, it was thought that the substrate binding to $\gamma$-secretase was preceded by an association of the substrate with an initial binding site, located in the proximity to the binding site^14^. Further investigation of the substrate binding by photo-affinity mapping found that the primary binding site is formed by PEN-2 and nicastrin, which is however distal to the active site formed by TM6 and TM7 of presenilin^15^. It was proposed that the substrate, by interacting with concomitant exosites, can move towards the active site either through the concave or the convex side of the horseshoe-shaped TMD of $\gamma$-secretase^15^. The early low-resolution structures of $\gamma$-secretase^16^ suggested that the binding path to the active site might lead through the inside of the horseshoe-shaped TMD. However, recently published cryo-EM structures of the $\gamma$-secretase complex with CTFs of both Notch and APP strongly indicate that substrate binding to the active site should proceed through the cleft between TM2 and TM6^17,18^. Indeed, the residues that were found to play an important role in the substrate binding are scattered at TM2, TM6 and TM9 of presenilin and form a putative last external binding site^19,20^. Such notion is also supported by coarse-grained simulations of $\gamma$-secretase with TMD of Notch and APP embedded in POPC membrane, in which the substrates were shown to finally dock to the surface formed by TM2, TM6 and TM9 of presenilin^21^.

Unfortunately, the mechanism of substrate recognition and recruitment remains elusive. Initially, it was suggested that nicastrin acts as the $\gamma$-secretase substrate receptor^22^. However, recent studies show that the main role of this subunit is to sterically block the binding of the substrates with large ectodomains^23^. This implies that the binding affinity is governed by the interactions between the substrate and enzyme transmembrane domains. Yet, $\gamma$-secretase binds and cleaves over 90 different substrates that do not share any sequence similarity^2,24^. Such diversity among $\gamma$-secretase substrates suggests that the driving forces for substrate binding are not based on specific residue-residue interactions.

$\gamma$-secretase localizes in liquid-ordered membrane microdomains enriched with cholesterol, often termed “lipid rafts”^25^. The enzyme activity was shown to be directly dependent on the membrane cholesterol content^26^. That is why statins have been investigated as a treatment for Alzheimer’s Disease; unfortunately, so far with mixed results^27–29^.

The mechanistic role of cholesterol in substrate proteolysis by $\gamma$-secretase also remains ambiguous. Initially it was thought that cholesterol binding by substrates might be involved in substrate presentation to the enzyme active site, as APP was shown to form weakly-bound binary complexes with cholesterol^30,31^; however, such hypothesis seems to be implausible as other $\gamma$-secretase substrates, e.g. Notch, do not show specific affinity for cholesterol^32^. This then leaves the question of how cholesterol participates in the cleavage process.

In this paper, we examine the role of cholesterol in substrate binding by $\gamma$-secretase, using the $\beta$-carboxyl-terminal fragment of APP ($\beta$-CTF), a direct precursor of $\beta$-amyloid polypeptides as a compelling example. We report that membrane thickening caused by cholesterol results in a negative hydrophobic mismatch between the membrane and both the substrate and the presenilin region formed by TM6 and TM9, localized in the immediate vicinity of the entrance to the active site. We show that $\beta$-CTF binding to this exosite is thermodynamically favorable in cholesterol-rich membranes and that cholesterol depletion abolishes the substrate binding. Finally, as a proof of concept, we directly show that, by increasing the membrane thickness independently of cholesterol, it is possible to restore the original high affinity for the $\beta$-CTF. Taken together, our results indicate that cholesterol-induced hydrophobic mismatch drives substrate association to the $\gamma$-secretase exosite formed by TM6 and TM9 of presenilin.

## Results and discussion

### Both $\gamma$-secretase and the substrate cause local membrane thinning in a cholesterol-rich membrane

**Figure 1:**
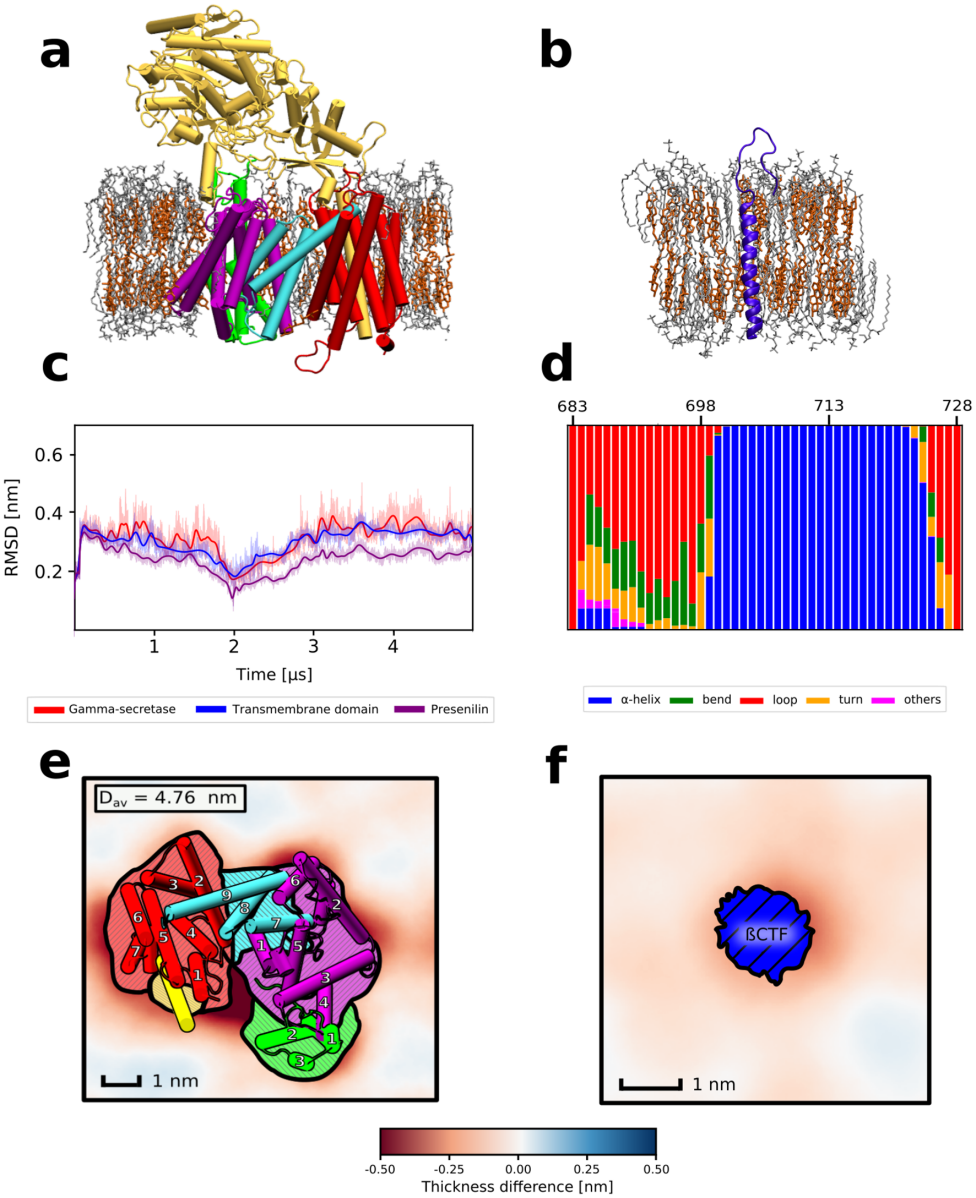
Representative structures of (**a**) $\gamma$-secretase or (**b**) $\beta$-CTF embedded in a cholesterol-rich DPPC membrane. Individual subunits of $\gamma$-secretase are indicated by colors: nicastrin (yellow), APH-1 (red), PEN-2 (green), presenilin NTF and CTF (purple and cyan, respectively). (**c**) Time evolution of the heavy-atom RMSD for $\gamma$-secretase (red), its transmembrane domain (blue) and presenilin (purple). (**d**) Populations of different types of secondary structures for $\beta$-CTF. Spatial variations in the average membrane thickness around (**e**) $\gamma$-secretase and (**f**) $\beta$-CTF. The colorscale depicts thinner (red) and thicker (blue) regions of the membrane than in the bulk. The helices are numbered in panel (**e**) according to their order in the $\gamma$-secretase subunits.

Membrane cholesterol content was previously shown to play an important role in activity of $\gamma$-secretase. To investigate how the protein-lipid interplay in cholesterol-rich bilayers affects $\gamma$-secretase and its substrate to facilitate the binding, we initially run conventional MD simulations for two systems: a single copy of either (1) $\gamma$-secretase complex or (2) its substrate $\beta$-CTF, embedded in a lipid bilayer composed of DPPC and cholesterol in 3:2 molar ratio (DPPC/Chl) that resembles the cholesterol-rich microdomains (Fig. 1a,b). Time evolution of root-mean-squared deviation (RMSD) of the protein heavy atoms of $\gamma$-secretase showed that the enzyme structure fluctuates around the state identified with cryo-EM (Fig. 1c) and neither TMD $\gamma$-secretase nor the whole enzyme undergo any major conformational change in a timescale of 5$\upmu \mathrm{s}$, in agreement with previous MD studies^33^. In case of $\beta$-CTF we observe that while the TMD region remains stable in a timescale of 1$\upmu \mathrm{s}$, the N-terminal helical fragment unwinds and forms an unstructured loop (Fig. 1d, region 683–699). The unwinding of $\beta$-CTF NTD in the presence of membrane cholesterol was also observed in previous MD studies^31,34^ and might reduce the steric repulsion between NTD domains of the substrate and nicastrin^23^. Overall, although we observe a conformational change of the $\beta$-CTF NTD in the presence of cholesterol that might reduce the repulsion between the substrate and the enzyme, no cholesterol-induced conformational transitions of $\gamma$-secretase are seen that could increase its affinity for the substrate binding.

With the aim to deepen the understanding of the impact of the membrane cholesterol on both the enzyme and the substrate, we analyzed the local properties of the DPPC/Chl membranes containing either $\gamma$-secretase or $\beta$-CTF and compared the results with the properties of the bulk DPPC/Chl membrane. Initially, we computed 2D profiles of the membrane thickness for the considered systems. As shown in Fig. 1e,f, both $\gamma$-secretase and $\beta$-CTF induce thinning of the cholesterol-rich membrane in their immediate proximity. Such protein-induced local thinning of the membrane suggests a negative mismatch between the TMD length of both the enzyme and the substrate versus the thickness of the membrane hydrophobic core, which might lead to the protein aggregation in the membrane^35^. What is particularly interesting is that the local membrane thinning in the system containing $\gamma$-secretase is especially visible in the proximity of the presenilin TM6 and TM9 (Fig. 1e), which have previously been suggested to participate in the substrate binding both by biochemical experiments^19,20^ and coarse-grained simulations^21^. Moreover, both $\beta$-CTF and presenilin TM6 and TM9 induce the decrease of deuterium order parameter values (S_CD_) of the lipids in their proximity with respect to the bulk membrane (Fig. S1), further indicating the hydrophobic mismatch between the DPPC/Chl membrane and both the substrate or the putative binding site of the enzyme.

Importantly, presenilin TM6 and TM9 are in the immediate vicinity of the cleft between presenilin TM2 and TM6, which has been previously suggested to govern the access to the enzyme active site^17,18^. We hypothesize that the local membrane thinning in the proximity of both $\beta$-CTF and TM6 and TM9 of presenilin might be a factor that promote the substrate binding to this region of $\gamma$-secretase.

### Membrane cholesterol content is critical for substrate association to the exosite of $\gamma$-secretase formed by presenilin TM6 and TM9

**Figure 2:**
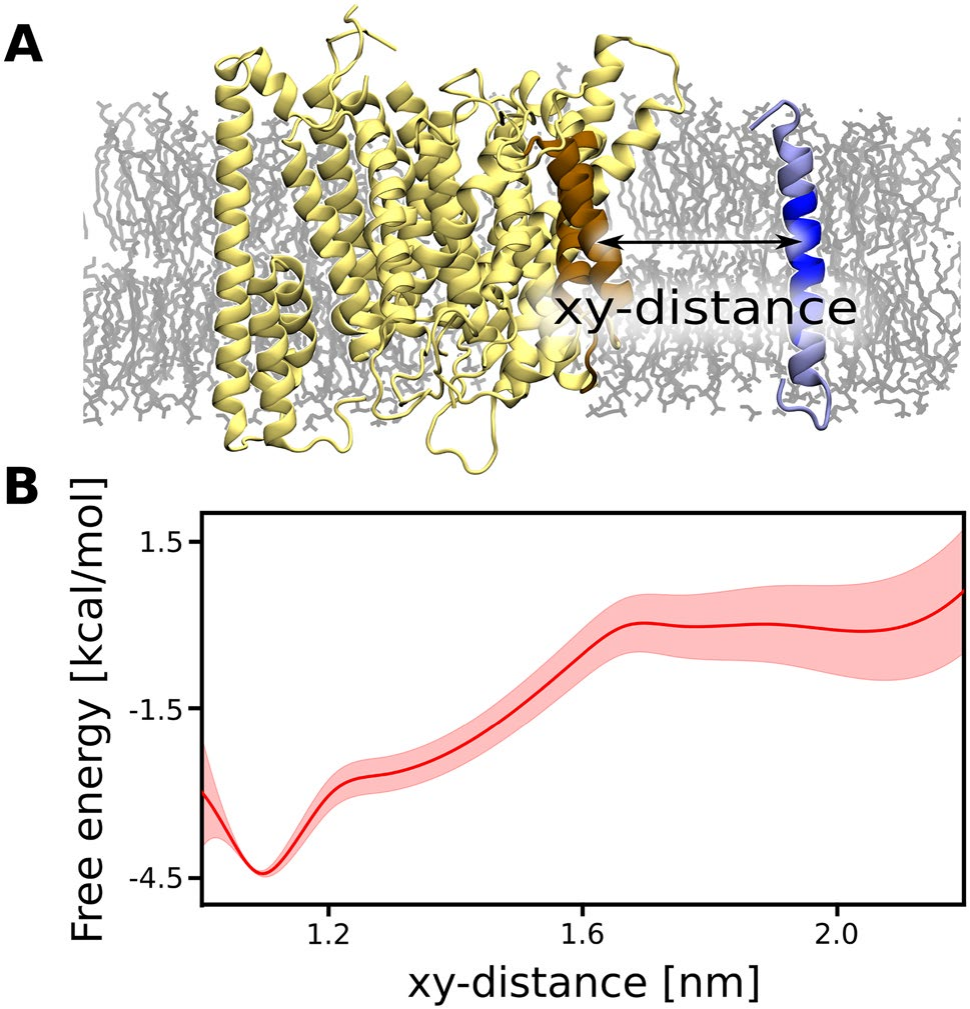
(**A**) Structural representation of the reaction coordinate used for our free energy simulations, i.e., center-of-mass distance projected on a membrane plane (*xy*-distance) between TM6 and TM9 of presenilin and TM of $\beta$-CTF. (**B**) The free energy profile for the substrate binding to the exosite of $\gamma$-secretase formed by presenilin TM6 and TM9.

To verify if the identified region of $\gamma$-secretase might play a role of the exosite in the substrate binding, we computed the free energy profile for the $\beta$-CTF association to the putative binding site formed by the presenilin TM6 and TM9 (*xy*-distance, Fig. 2A) using the umbrella sampling method. The N-terminal domain of nicastrin was previously shown to exclude the substrates with large ectodomains, while it was not necessary for the substrate affinity^23^ nor the stability of $\gamma$-secretase TMD^33^. Thus, to accelerate the sampling of the binding process, we removed N-terminal domains of both nicastrin and the substrate to reduce the size of the system. Notably, the membrane thinning in the proximity of $\gamma$-secretase was not affected by removal of the nicastrin N-terminal domain (Fig. S2).

The resulting free energy profile (Fig. 2B) exhibits a broad free energy well with a 4.5 kcal mol$^-1$ deep minimum at *xy*-distance of 1.1 nm, corresponding to the substrate intimately interacting with the presenilin TM6 and TM9 and the overall affinity estimated to −3.5(8) kcal mol$^-1$. In contrast to the favorable substrate binding to TM6 and TM9, the substrate association to another membrane-thinning region of $\gamma$-secretase, inside the horseshoe-shape of TMD formed by PS-1, PEN-2 and APH-1, is associated with a free energy penalty up to 4 kcal mol$^-1$ (Fig. S3, middle and bottom panels). These results strongly support the notion that the substrate binds to the active site through the concave side of the horseshoe-shaped TMD of $\gamma$-secretase and that the identified binding site plays a pivotal role during substrate recruitment. The non-zero slope of the free energy profile reaches up to 1.7 nm, which is unexpected for protein-protein binding dominated by non-polar interactions. We speculate that the substrate association to $\gamma$-secretase might be induced by the negative hydrophobic mismatch between the membrane and both the substrate and the binding site.

**Figure 3:**
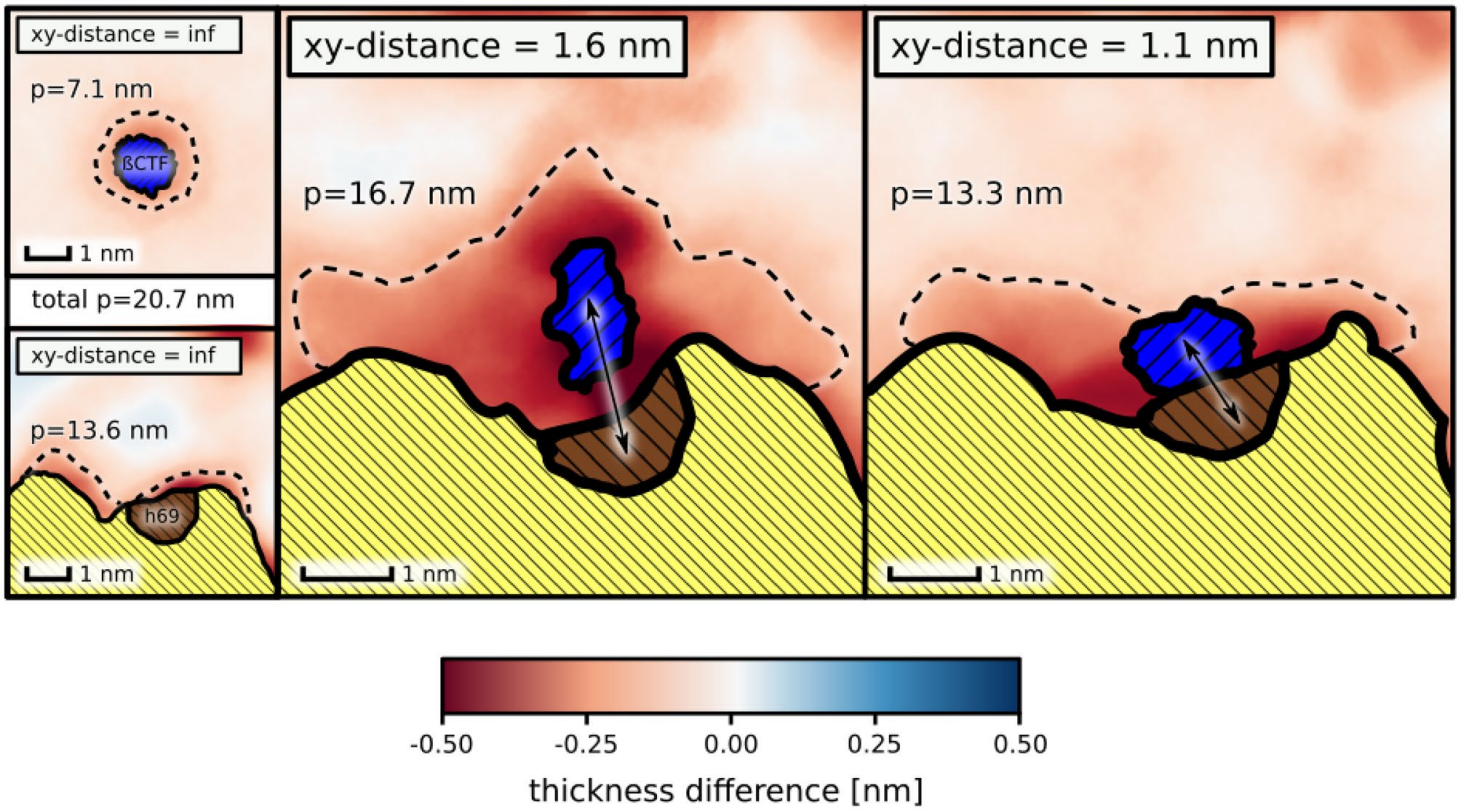
Spatial variations in the average membrane thickness in proximity of the $\gamma$-secretase exosite and $\beta$-CTF: isolated (left, *xy*-distance = $inf$), at the slope of the free energy profile for the substrate binding (middle, *xy*-distance = 1.6 nm) and in complex (right, *xy*-distance = 1.1 nm). The perimeter (p) of the membrane regions thinned by the proteins is represented with dashed lines.

With aim to examine the role of hydrophobic mismatch in the substrate binding, we computed the 2D profile of the membrane thickness for the identified $\gamma$-secretase complex with the substrate and compared it with the thickness profiles obtained for the isolated proteins embedded in the DPPC/Chl membrane (Fig. 3). Since the energetic penalty due to negative mismatch is proportional to the perimeter of the membrane area perturbed by the inclusion (here, by the proteins)^36^, it is clearly visible that upon complex formation this penalty is reduced as the thinner areas of the membrane largely overlap around the bound state. Assuming that these thinner regions can be treated as a disordered phase distinct from the bulk liquid-ordered membrane phase, we can roughly estimate the contribution of the hydrophobic mismatch to the binding free energy by multiplying the length of the phase boundary by the line tension between the phases. In our case, the membrane region was defined as disordered when it was thinner by at least 0.15 nm than the bulk membrane. The computed perimeters of the disordered membrane regions in the proximity of the isolated proteins were estimated to 13.6 and 7.1 nm for the $\gamma$-secretase exosite and the substrate, respectively (dashed line in Fig. 3). For the complex of $\gamma$-secretase with the substrate, the perimeter of the disordered region was approximately of 13.3 nm, which is smaller by 7.4 nm with respect to the isolated proteins. Since the experimental values of the line tension between the ordered and disordered phases vary between 1 and 3 pN^37^, we find that complex stabilization due to hydrophobic mismatch is in the range −1.1 to −3.2 kcal mol$^-1$, which accounts for a substantial fraction of the overall binding affinity (− 3.5(8) kcal mol$^-1$) estimated with the free energy profile. Similarly, at the *xy*-distance corresponding to the slope of the free energy profile ($\sim$ 1.6 nm), the perimeter of the disordered region is reduced by 4.0 nm to16.7 nm, which corresponds to the free energy gain between −0.6 kcal mol$^-1$ and −1.7 kcal mol$^-1$. This result shows that hydrophobic mismatch could be also responsible for the observed long-range lipid-mediated attraction between the binding partners.

### Cholesterol depletion abolishes substrate association to the exosite of $\gamma$-secretase formed by presenilin TM6 and TM9

**Figure 4:**
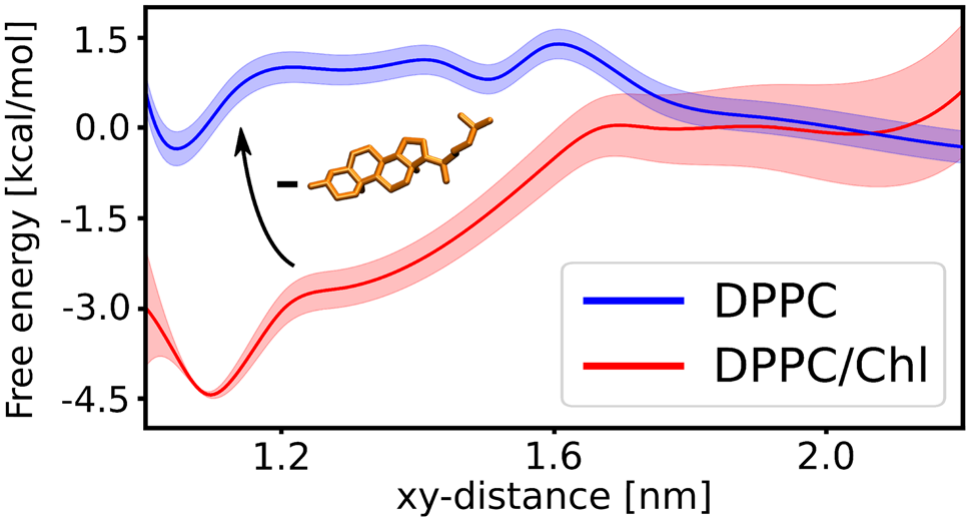
The free energy profile for the substrate binding by $\gamma$-secretase in the presence (red) or absence (blue) of cholesterol in DPPC membrane.

To verify the role of cholesterol in binding the $\beta$-CTF to $\gamma$-secretase, we performed US simulations for the substrate binding in pure DPPC membrane, using the same reaction coordinate as previously. As shown in Fig. 4, the depletion of membrane cholesterol abolished the affinity of $\gamma$-secretase to bind the substrate, as indicated by a very shallow bound-state minimum in the obtained free energy profile and the overall unfavorable free energy change of 0.4(4) kcal mol$^-1$ associated with the binding.This result is in line with previous experimental observations, where cholesterol removal abolished the substrate cleavage by $\gamma$-secretase^26^. To propose a rationale for the abrogated binding in the absence of cholesterol, we performed additional conventional MD simulations of either $\beta$-CTF or $\gamma$-secretase embedded in the pure DPPC membrane.

**Figure 5:**
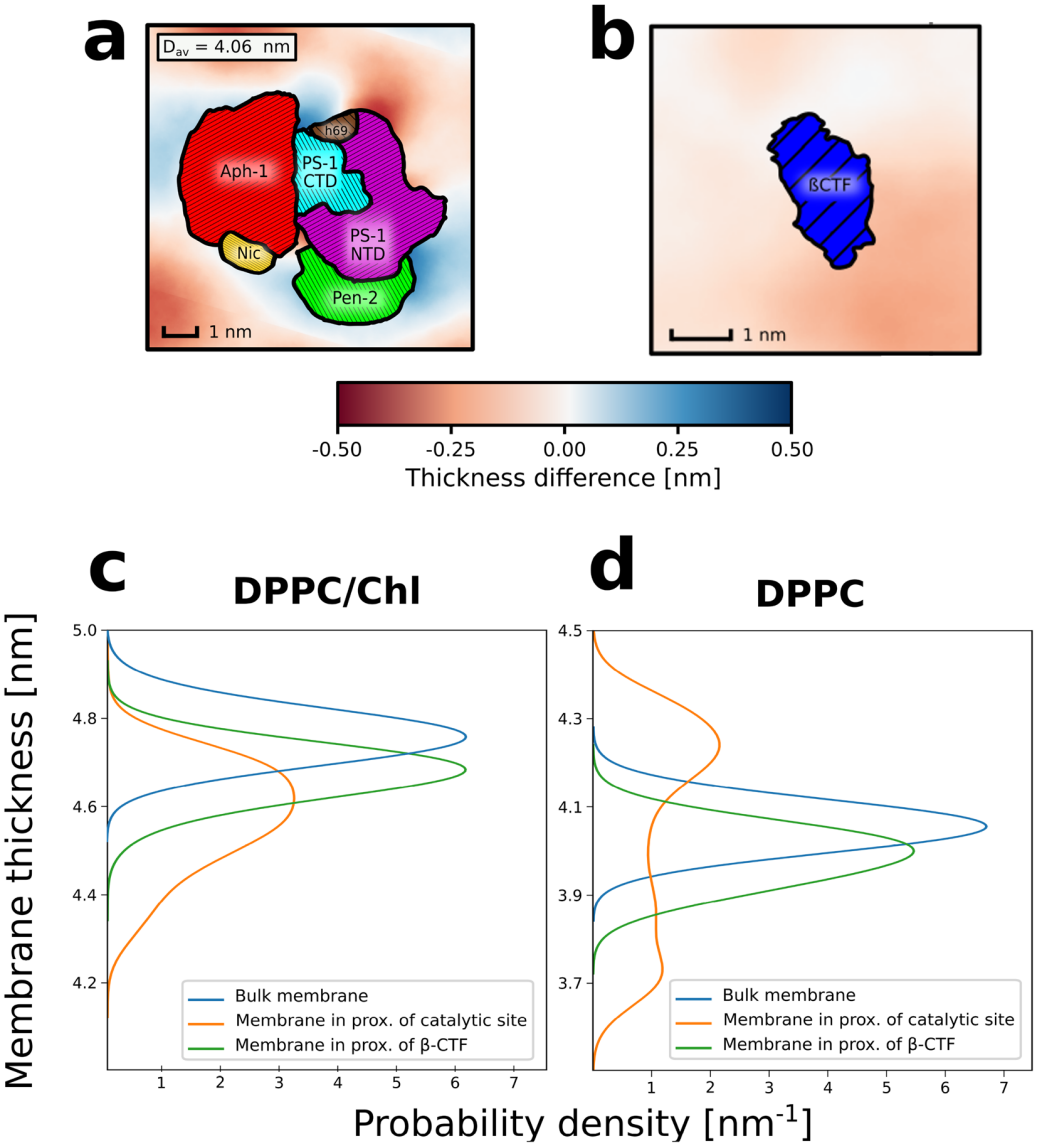
2D profiles of the membrane thickness for (**a**) $\gamma$-secretase and (**b**) $\beta$-CTF embedded in pure DPPC membrane. Probability distributions of the membrane thickness in the proximity of initial substrate binding site of $\gamma$-secretase (orange), $\beta$-CTF (green) or in the bulk (blue) for cholesterol-rich (**c**) or pure (**d**) DPPC membrane.

The analyses of local membrane properties for DPPC membrane showed a local increase of the membrane thickness in the proximity of the $\gamma$-secretase exosite (Fig. 5a,d), suggesting that the enzyme induces the ordering of neighbouring lipids. On the other hand, the lipids that are close to the substrate are more disordered, which is shown by both the induced thinning of the membrane and the reduced S_CD_ parameters for the neighbouring lipids (Fig. 5b,d and Fig. S4). Contrarily to the DPPC/Chl membrane, where the negative mismatch was observed for both the substrate and the enzyme (Fig. 5c and Fig. S1), in DPPC membrane, the proteins have an opposite impact on the local membrane properties. Considering that cholesterol depletion does not induce any significant conformational changes in the proteins (Fig. S5 and Fig. S6) and the negligible affinity of $\gamma$-secretase to the substrates in the absence of cholesterol, these results suggest that the cholesterol-induced negative mismatch plays a critical role in the substrate binding by $\gamma$-secretase.

### Hydrophobic mismatch is a major driving force for substrate-binding by $\gamma$-secretase

Finally, we aimed to examine if indeed the main role of cholesterol in the substrate binding by $\gamma$-secretase is to induce a negative hydrophobic mismatch between the membrane and the proteins. For this purpose, we re-computed the free energy profile for the substrate binding by the $\gamma$-secretase exosite this time in the pure DPPC membrane with the imposed thickness matching that of DPPC/Chl system. As our initial trials with the DPPC membrane failed due to the membrane phase transitions induced by the membrane-thickening potential, we switched to a membrane composed of SOPC lipids. SOPC membrane has two major advantages—the membrane thickness is comparable to the DPPC membrane^38^, while it also has moderately low phase transition temperature (6.7 °C)^39^ and therefore the membrane-thickening potential does not induce phase transitions as in case of DPPC.

**Figure 6:**
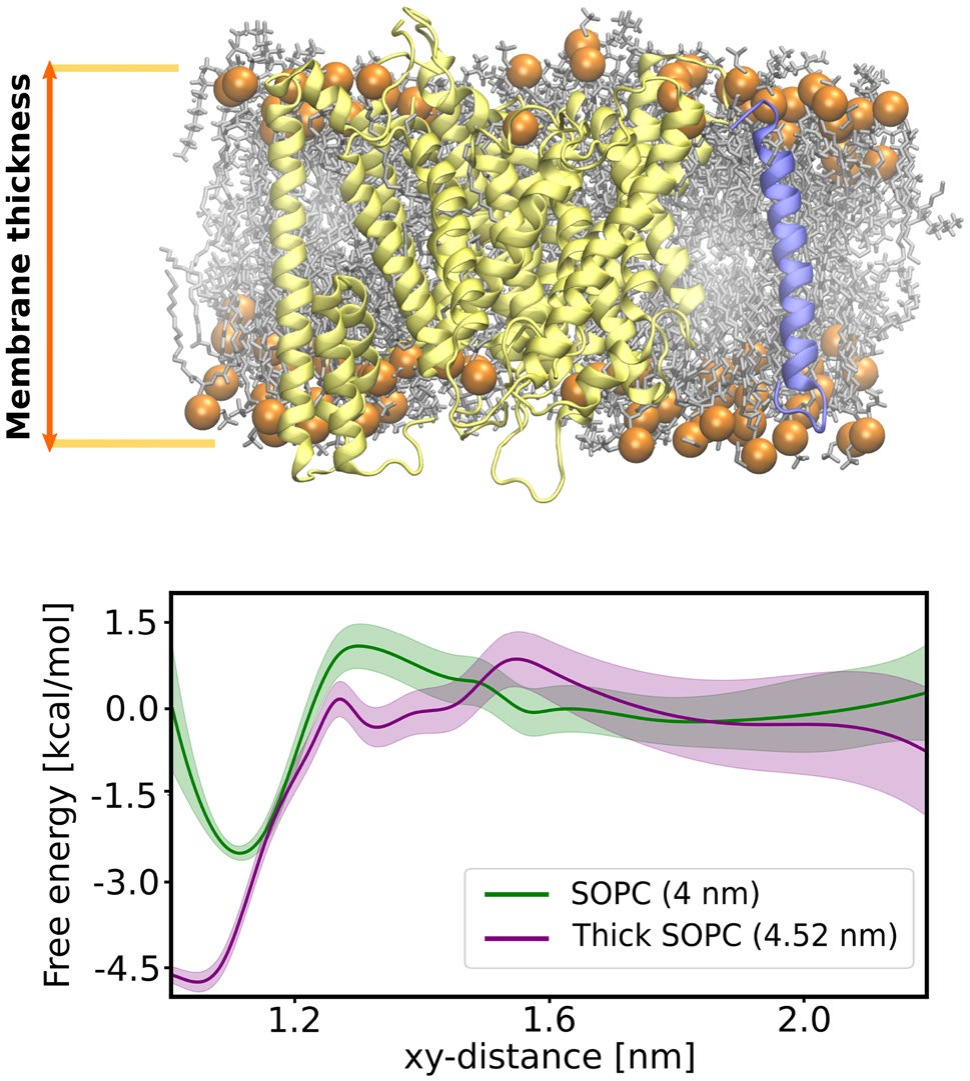
(Top) Structural representation of the membrane thickness definition. (Bottom) The free energy profiles for the substrate binding to $\gamma$-secretase in the SOPC membrane (green) and in the SOPC membrane with the imposed thickness of DPPC/Chl membrane (purple).

We performed two sets of US simulations for the substrate binding to the $\gamma$-secretase exosite: in the first one, we did not apply any additional potential; in the second, we used additional potential to increase the membrane thickness to 4.52 nm (thick SOPC), which is the average thickness for the DPPC/Chl membrane containing both $\gamma$-secretase and the substrate. As shown in Fig. 6, the depth free energy minimum associated with the substrate binding by $\gamma$-secretase increases from 2.5 kcal mol$^-1$ in the SOPC membrane to 4.5 kcal mol$^-1$ in the thick SOPC membrane and the free energy of binding increases from −1.1(3) kcal mol$^-1$ to −3.1(7) kcal mol$^-1$, respectively. Noteworthily, the non-zero slope of the free energy profile reaches up to 1.5 nm in thick SOPC membrane, suggesting a similar recognition mechanism as in the DPPC/Chl membrane. Overall, these results further support the hypothesis that substrate binding by $\gamma$-secretase is driven by the negative hydrophobic mismatch.

## Conclusions

In this work, we described the role of cholesterol in substrate recognition by $\gamma$-secretase on the example of Amyloid Precursor Protein $\beta$-CTF by means of MD simulations. Initially, we showed that the thickness of DPPC/Chl membrane is reduced in the proximity of both the substrate and the putative binding site formed by the presenilin TM6 and TM9. In line with previous studies^17–21^, our free energy simulations suggest that the substrate binding pathway to the active site leads through the concave side of the horseshoe-shaped TMD of $\gamma$-secretase and that the identified exosite play an important role in the substrate recruitment. Consistently with previous experimental findings, we observed that cholesterol depletion from DPPC/Chl membrane abolished the $\gamma$-secretase affinity for substrates. We found that in the pure DPPC membrane $\gamma$-secretase and its $\beta$-CTF substrate have an opposite effect on the membrane properties, suggesting that the substrate affinity of $\gamma$-secretase is driven by the hydrophobic mismatch. Finally, we directly show in the SOPC membrane that the increased membrane thickness enhances the propensity of $\gamma$-secretase to bind substrates. Based on study in SOPC membrane we also point out that the dominant role of the hydrophobic mismatch in the substrate recognition by $\gamma$-secretase can be experimentally verified by assessing how the enzyme proteolytic activity changes with the increase of the membrane lipids length. Overall, our results provide evidence that the hydrophobic mismatch is a driving force for the substrate recognition and binding by $\gamma$-secretase.

## Methods

### Simulation protocol

All molecular dynamics (MD) simulations were performed using GROMACS 5.1.4^40^ with the PLUMED 2.2.3 plugin^41^. CHARMM36 force field^42^ was used for the proteins and lipids and the TIP3P model was used to represent water. The simulations were carried out in the isothermal-isobaric (NPT) ensemble using periodic boundary conditions. The constant temperature was kept at 320 K using the Nosé–Hoover thermostat and the pressure was maintained at 1 bar semi-isotropically in the plane of the bilayer and perpendicular to the bilayer using Parrinello–Rahman algorithm. Electrostatic interactions were calculated using Particle Mesh Ewald (PME) method with a real-space cut-off of 1.2 nm. Van der Waals interactions were evaluated using a smooth cut-off of 1.2 nm with a switching distance of 1 nm. Bond lengths were constrained using the SHAKE algorithm. The equations of motion were integrated using a leap-frog algorithm with a time step of 2 fs. All of the simulated systems were built using the CHARMM-GUI Membrane Builder server^43^. In each of the simulated systems, K$^+$ and Cl$^-$ ions were added to maintain the physiological ionic strength (0.15 M). Minimization and equlibration of the systems were performed following the default CHARMM-GUI Membrane Builder protocol^43^.

### Conventional MD simulations

The whole $\gamma$-secretase complex systems were composed of a single copy of $\gamma$-secretase (pdb ID 5FN2)^44^ in a 10.5 nm$~\times$ 10.5 nm $\times$ 19 nm rectangular box, embedded in a membrane composed of either 240 DPPC and 160 cholesterol molecules (DPPC/Chl system) or 370 DPPC molecules (DPPC system) solvated with approx. 50,000 water molecules. 5 $\upmu \mathrm{s}$ trajectory was obtained for each system. $\beta$-CTF systems were composed of a single copy of the protein (pdb ID 2LP1)^30^ in a 7.2 nm $\times$ 7.2 nm $\times$ 9.2 nm rectangular box embedded in a membrane composed of either 108 DPPC and 72 cholesterol molecules (DPPC/Chl system) or 150 DPPC molecules (DPPC system), solvated with approx. 8000 water molecules. For each system, 1 $\upmu \mathrm{s}$-long trajectory was obtained. Bulk membrane systems were composed of either 100 DPPC and 66 cholesterol molecules (DPPC/Chl system) or 120 DPPC molecules (DPPC system), placed in 6 nm $\times$ 6 nm $\times$ 7.5 nm rectangular box and solvated with approx. 4500 water molecules. For each system, 0.5 $\upmu \mathrm{s}$-long trajectory was obtained.

### Free energy simulations

The free energy profile associated with the substrate binding to the $\gamma$-secretase exosite formed by TM6 and TM9 of presenilin in the presence or the absence of cholesterol was computed with the replica-exchange umbrella sampling (REUS) method^45^. The reaction coordinate (xy-distance) was defined as the distance between the centers of mass (COM) of backbone atoms of the selected transmembrane fragment of $\beta$-CTF (residues 706–718) and of the presenilin TM6 and TM9 (residues 240–262 and 430–451, respectively). The N-terminal domain of nicastrin (residues 1–662) and the N-terminal portion of $\beta$-CTF (residues 683–694) were removed to reduce the system size, and thus to allow for the better convergence of the free energy profiles. Finally, the simulated systems were composed of a truncated $\gamma$-secretase and $\beta$-CTF in a 10.5 nm $\times$ 10.5 nm $\times$ 19 nm rectangular box, embedded in a bilayer composed of 192 DPPC and 128 cholesterol molecules, solvated with approx. 25,000 water molecules. As shown for 1 $\upmu \mathrm{s}$-long MD simulation of the truncated $\gamma$-secretase Fig. S2, the spatial variation of the average membrane thickness around the enzyme was not affected by the removal of the nicastrin N-terminal domain.

The initial configurations for REUS simulations in DPPC/Chl system were obtained with 200 ns-long steered-MD simulation, during which the the center of one-sided harmonic potential with a spring constant of 5000 KJ mol$^-1$ nm$^-2$ was moved with a constant velocity from the initial value of 2.5–1 nm along the *xy*-distance. Additionally, during the steered-MD simulation the root-mean-square deviation (RMSD) based restraint with a spring constant of 500 KJ mol$^-1$ nm$^-2$ was applied to the backbone conformation of each $\beta$-CTF fragment and presenilin TM6 and TM9 to prevent any conformational changes induced by the harmonic potential. The initial frames for the pure DPPC system were obtained by removal of the cholesterol from the initial frames for DPPC/Chl windows and subsequent 100 ns-long equilibration. Two sets of 8 equally spaced US windows were used, spanning the ranges 1.05–1.75 nm and 1.75–2.45 nm of the reaction coordinate, respectively. The spring constant of the harmonic biasing potential was set to 3500 KJ mol$^-1$ nm$^-2$ in each REUS window. Each of the REUS windows were simulated for 1.75 $\upmu \mathrm{s}$ and free energy profiles were determined using the weighted histogram analysis method (WHAM; see details below)^46^.

The same reaction coordinate was used for the REUS simulations of substrate binding to $\gamma$-secretase in the SOPC membrane. Here, the system was composed of a single copy of a truncated $\gamma$-secretase with the substrate in a 10.5 nm $\times$ 10.5 nm $\times$ 19 nm rectangular box, embedded in a membrane composed of 274 SOPC molecules and solvated with approx. 20,000 water molecules. The initial frames for REUS simulations were obtained with 200 ns of steered-MD simulations, in which the center of the one-sided harmonic potential with a spring constant of 5000 KJ mol$^-1$ nm$^-2$ was moved from 1 to 2.4 nm along the *xy*-distance, starting from the substrate-enzyme complex obtained during REUS simulations in the DPPC/Chl membrane. In the case of the SOPC membrane with increased thickness (thick SOPC), the steered-MD simulation was preceded by additional 100 ns simulation run, in which the center of harmonic potential with a force constant of 8000 KJ mol$^-1$ nm$^-2$ was used to increase the distance along the membrane normal between COM of phosphorus atoms from 4 to 4.52 nm (which was the average distance between the phosphorus atoms of the opposing leaflets computed for $\gamma$-secretase with a substrate embedded in DPPC/Chl). The same restraint was further used to keep the SOPC membrane thickness at 4.5 nm during REUS simulations. Two sets of 7 equally spaced US windows were used, spanning the ranges 0.95–1.55 nm and 1.55–2.15 nm of the reaction coordinate, respectively. The spring constant of the harmonic biasing potential was set to 3500 KJ mol$^-1$ nm$^-2$ in each REUS window. Each of the REUS windows were simulated for 1 $\upmu \mathrm{s}$.

The initial configurations for REUS simulations of the substrate association to the interior the horseshoe-shape of $\gamma$-secretase TMD formed by PS-1, PEN-2 and APH-1 in DPPC/Chl membrane were obtained with steered-MD simulation. Here, we started from the same frame as in the steered-MD for the substrate binding to TM6 and TM9 of presenilin. As a reaction coordinate, a COM distance between the $\beta$-CTF backbone atoms (residues 706–718) and of the presenilin TM1 (residues 78–99) and APH-1 TM1 (residues 2–28). The reaction coordinate is represented on the top panel of Fig. S3. During the 400 ns-long steered-MD run, the center of one-sided harmonic potential with a spring constant of 5000 KJ mol$^-1$ nm$^-2$ was moved with a constant velocity from the initial value of 3.6–1 nm along the reaction coordinate. Two sets of 8 equally spaced US windows were used, spanning the ranges 0.95–1.65 nm and 1.65–2.35 nm of the reaction coordinate, respectively. Each of the REUS windows were simulated for 1 $\upmu \mathrm{s}$.

The uncertainties of the free energy profiles were computed as follows: initially, the convergence of the free energy profiles was examined to exclude from the analysis the portion of the trajectory corresponding to the relaxation of the initially prepared configurations. In case of DPPC, SOPC and thick-SOPC systems, the free energy profiles converge with the removal of the initial 200 ns from each window (Fig. S7, Fig. S8 and Fig. S9), while in the case of DPPC/Chl systems the profiles converge with the removal of 300 ns and 500 ns for the interior of the $\gamma$-secretase horseshoe-shape and TM6-TM9 binding sites, respectively (Fig. S10 and Fig. S11). These data were excluded from further analysis. Next, for the remaining data, the time series autocorrelation of the reaction coordinate (*xy*-distance) were examined in each US window. These autocorrelation times were then used to compute the number of uncorrelated points to be generated per each fake data sets of the Monte Carlo bootstrap error analysis (the details of the method are described in the SI text).

### Trajectory analysis

The conformational variability of $\gamma$-secretase in DPPC/Chl and DPPC membranes was quantified using RMSD of the position of protein heavy atoms, which was computed using gmx rms from the GROMACS package^40^. The secondary structure of $\beta$-CTF was assigned with a dictionary of protein secondary structure (DSSP) method proposed by Kabsch and Sander^47^, implemented in the python library MDTraj^48^.

2D profiles of the membrane thickness for the membranes containing $\gamma$-secretase or/and $\beta$-CTF were calculated using g_lomepro^49^. For this analysis, we used the trajectories from either conventional MD simulations (for the isolated proteins) or US simulation (for the proteins associated with each other) with a frame spacing of 0.5 ns. With g_lomepro, the positions of the phosphorus atoms of upper and lower leaflets with respect to the *z*-axis were mapped on two *xy*-grids composed of 500 $\times$ 500 points. The average thickness was then computed as a distance between the points on the grid of the upper leaflet and lower leaflet. The average volume occupied by either $\gamma$-secretase or $\beta$-CTF in the simulation box was computed using gmx densmap.

The perimeters of the membrane areas perturbed by the proteins were computed using the results obtained with the g_lomepro and gmx densmap tools. The perturbed areas were defined as a regions with a membrane thickness lowered by at least 0.15 nm with respect to the bulk membrane (4.76 nm) that was not occupied by the proteins. OpenCV python library^50^ was used to compute the perimeters of the perturbed membrane regions. In case of $\beta$-CTF, the perimeter was computed for the interface of the disordered region and the bulk membrane. In case of $\gamma$-secretase, the perimeter included both the perimeter of the interface between the disordered region-bulk interface and the interface between the protein and the disordered membrane region. The change of the perturbed perimeter area length was computed by subtracting the perimeter for the isolated proteins from the perimeters obtained for the initially associated proteins (*xy*-distance = 1.6 nm) or the protein complex (*xy*-distance = 1.1 nm). The perimeter of the membrane-protein interface remains roughly constant for the isolated $\gamma$-secretase and for $\gamma$-secretase in complex with the substrate, and thus its contribution to the perimeter change can be neglected. The contribution of the hydrophobic mismatch to the binding free energy was estimated as the change of the perimeter length times as the experimental values of the line tension $\mathrm{({1}-{3}~pN}$^36^).

The comparison of the protein impact on the lipid acyl chain ordering was performed by computing deuterium order parameter (*S*_*CD*_), defined as $S_{CD} = 0.5 \left\langle 3 \cos ^2 \theta -1 \right\rangle$, in the proximity of $\gamma$-secretase, $\beta$-CTF and in the bulk membrane. For this purpose, a home made TCL script was used to compute $S_{CD}$ for the lipids whose COM was closer than 1.6 nm in the membrane plane to COM of either $\beta$-CTF or TM6 and TM9 of presenilin (in the conventional MD simulations with either of proteins) or for the lipids in the bulk membrane. To assess the uncertainty of the *S*_*CD*_ parameters, computed the *S*_*CD*_ parameters for the independent 100 ns long trajectory blocks and estimated the uncertainty as a standard deviation of the mean *S*_*CD*_ value.

All of the molecular images were created using VMD^51^.

## Supplementary Information

Supplementary Figures
