# Supplementary material for: Role of cholesterol in substrate recognition by γ-secretase
Source: Sci Rep. 2021 Jul 26;11:15213. doi: 10.1038/s41598-021-94618-2 (PMC8313713; doi:10.1038/s41598-021-94618-2)
Supplement: Supplementary file 1 — Supplementary Figures [file 41598_2021_94618_MOESM1_ESM.pdf]

Supplementary Information

Role of cholesterol in substrate recognition by γ–secretase

Łukasz Nierzwicki[1,*], Michał Olewniczak[1], Paweł Chodnicki[1], and Jacek Czub[1]

[1]Department of Physical Chemistry, Gdansk University of Technology, Gdansk, 80-233, Poland
[*]nierzwicki.l@gmail.com

Uncertainties of the free energy profiles.

To compute uncertainties of the free energies profiles, we performed bootstrap error analysis. First, since the original time-series data generated in the umbrella sampling (US) simulations are not statistically independent, we calculated the autocorrelation times of each of the US-generated time series of the reaction coordinate. Next, we estimated the number of uncorrelated data samples, N, in each of these US windows separately, by dividing the number of points in a time series by the corresponding autocorrelation time (expressed in in the units of time intervals between original points). Next we generated 200 sets of bootstrap time series for each of the US windows by picking at random (with replacement) N data samples from the original US-generated distributions. Technically, this was done by uniformly sampling the corresponding cumulative distribution functions in the [0,1] interval (i.e., through inverse transform sampling). For each set of “fake” time series, we then performed the WHAM procedure in the same way as for the original data. The standard deviation of the average free energies obtained from the “fake” data sets is an estimate for the statistical uncertainty of the free energies computed using the original data.

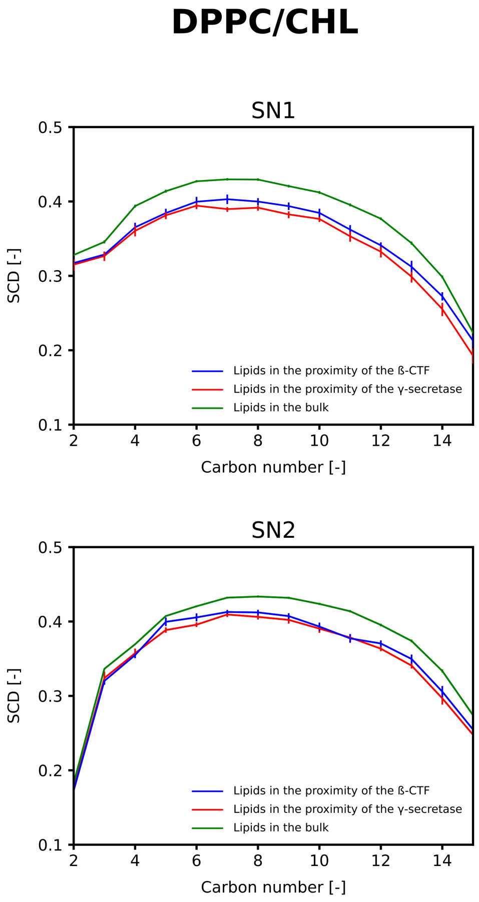

Figure S1. Deuterium order parameters (SCD) computed for the lipids in the proximity of γ–secretase substrate binding site (orange), β–CTF (blue) or in the bulk (green) in cholesterol–rich DPPC membrane. The lipids were accounted as close to the proteins when the COM distance in the membrane plane was smaller than 1.6 nm to β–CTF or to TM6 and TM9 of presenilin.

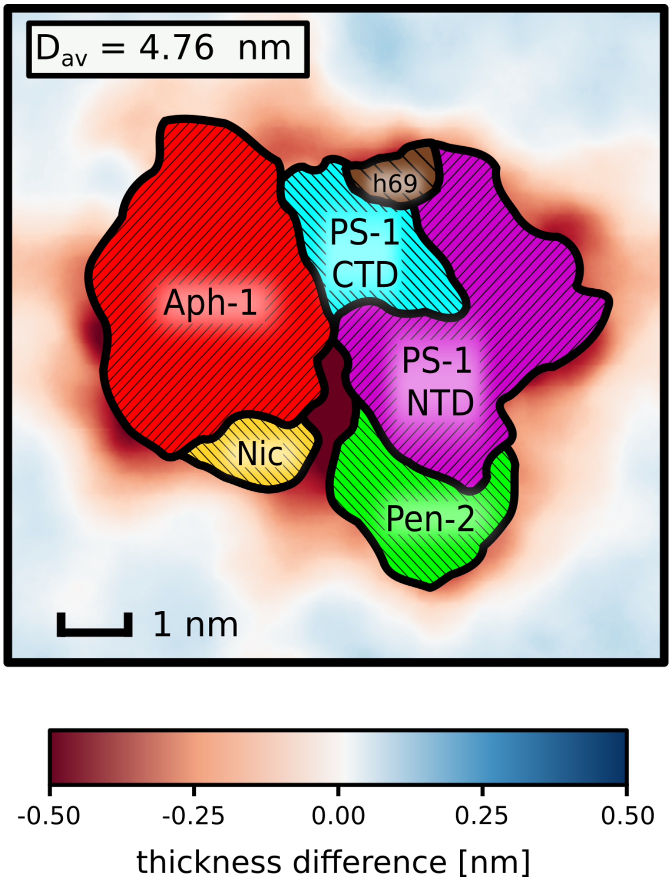

Figure S2. Spatial variations in the average membrane thickness around γ–secretase with removed N–terminal domains of nicastrin. The colorscale depicts thinner (red) and thicker (blue) regions of the membrane than in the bulk.

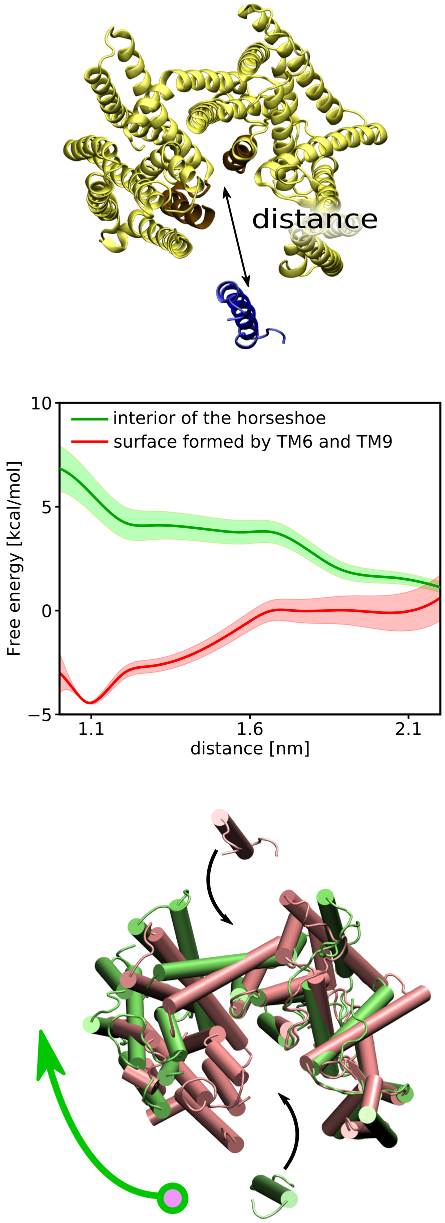

Figure S3. (top) Structural representation of the reaction coordinate for the substrate association to the interior the horseshoe–shape of γ–secretase TMD, namely the center-of-mass distance projected on a membrane plane (distance) between TM of β–CTF and of presenilin TM1 and APH–1. (middle) The free energy profiles for the substrate association to the horseshoe–shape of γ–secretase TMD (green) and to the TM6 and TM9 of presenilin (red). (bottom) The conformational state of the γ–secretase TMD with the substrate bound to either TM6 and TM9 of presenilin (pink) or to the interior of the TMD horseshoe–shape of γ–secretase (green). The interior of γ–secretase horseshoe–shape is to narrow to accomodate the substrate, and thus the substrate binding to this region induces an unfavorable stretching of the γ–secretase TMD (green arrow). The energetic penalty of this extensive conformational change probably overcomes the free energy gain associated with the substrate reaching the thinned area of the membrane inside the horseshoe–shape, resulting in overall repulsive interaction between the interior of the horseshoe–shape and the substrate.

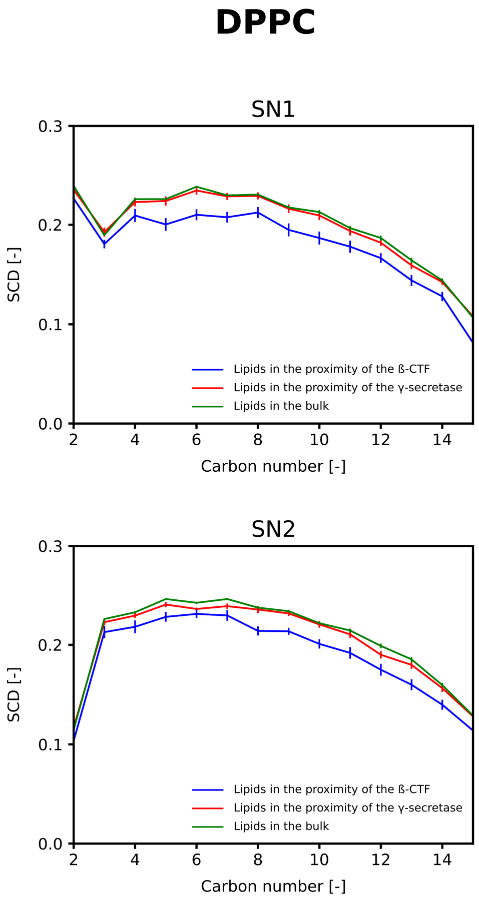

Figure S4. Deuterium order parameters (SCD) computed for the lipids in the proximity of γ–secretase substrate binding site (orange), β–CTF (blue) or in the bulk (green) in pure DPPC membrane. The lipids were accounted as close to the proteins when the COM distance in the membrane plane was smaller than 1.6 nm to β–CTF or to TM6 and TM9 of presenilin.

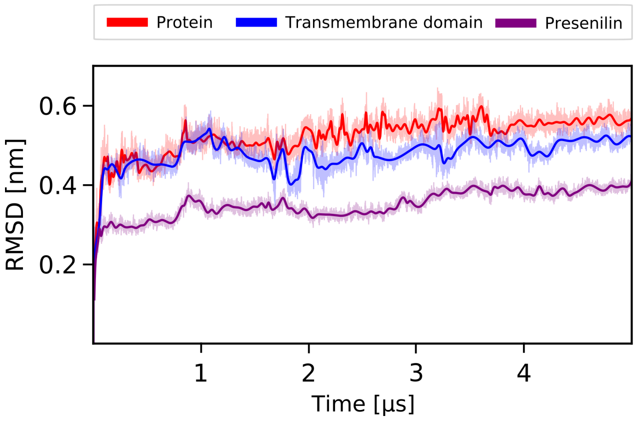

Figure S5. Time evolution of RMSD for γ–secretase (red), its transmembrane domain (blue) and presenilin (purple) in DPPC membrane.

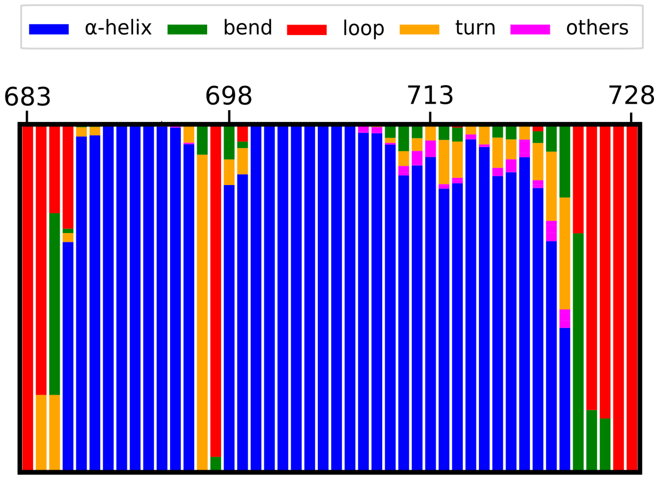

Figure S6. Populations of different types of secondary structures for β–CTF embedded in DPPC membrane.

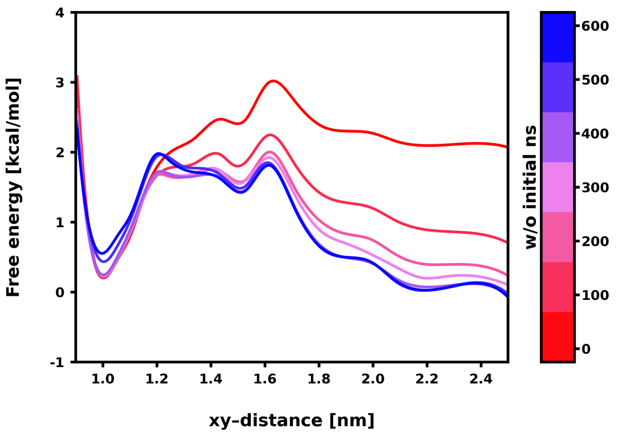

Figure S7. Convergence of the free energy profile for the substrate binding to presenilin TM6 and TM9 in DPPC membrane. The colorscale represents the amount of the discarded trajectory from the beginning of the simulation, ranging from 0 ns (red) to 600 ns (blue).

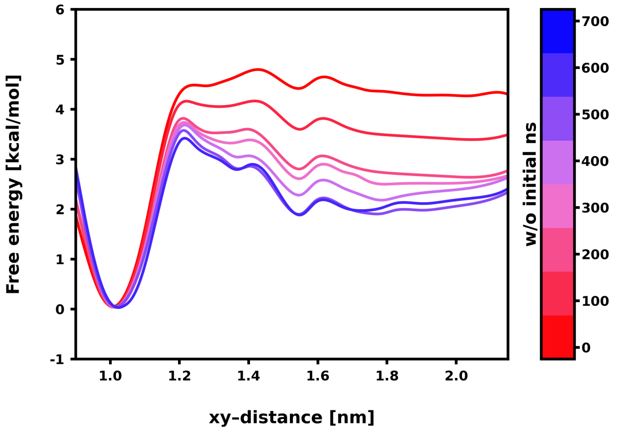

Figure S8. Convergence of the free energy profile for the substrate binding to presenilin TM6 and TM9 in SOPC membrane. The colorscale represents the amount of the discarded trajectory from the beginning of the simulation, ranging from 0 ns (red) to 700 ns (blue).

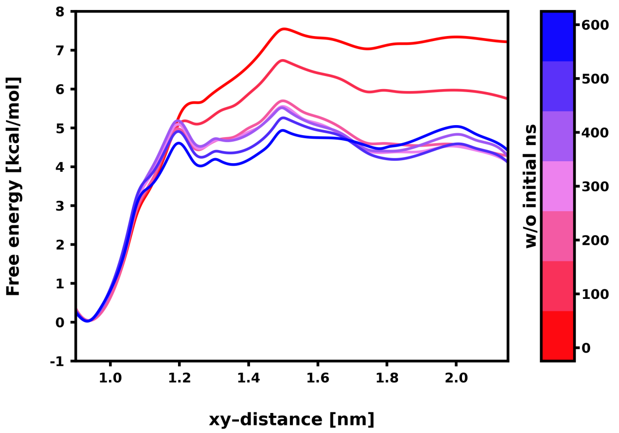

Figure S9. Convergence of the free energy profile for the substrate binding to presenilin TM6 and TM9 in SOPC membrane with increased thickness (thick SOPC). The colorscale represents the amount of the discarded trajectory from the beginning of the simulation, ranging from 0 ns (red) to 600 ns (blue).

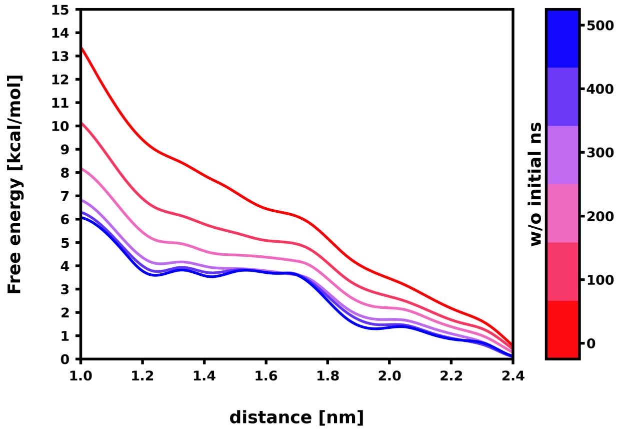

Figure S10. Convergence of the free energy profile for the substrate binding to the interior of γ–secretase horseshoe in DPPC/Chl membrane. The colorscale represents the amount of the discarded trajectory from the beginning of the simulation, ranging from 0 ns (red) to 500 ns (blue).

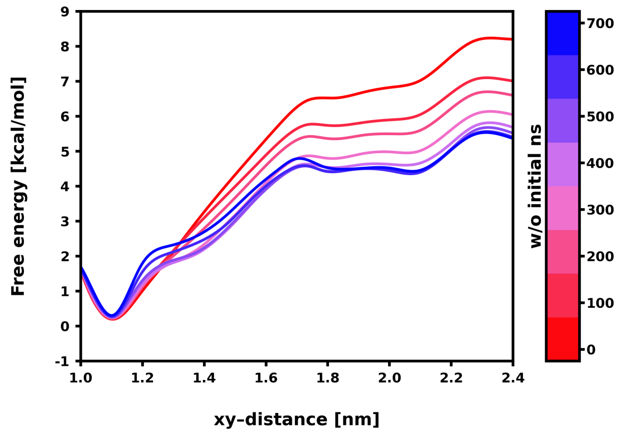

Figure S11. Convergence of the free energy profile for the substrate binding to presenilin TM6 and TM9 in DPPC/Chl membrane. The colorscale represents the amount of the discarded trajectory from the beginning of the simulation, ranging from 0 ns (red) to 600 ns (blue).
